# A *Drosophila* model for mechanistic investigation of tau protein spread

**DOI:** 10.1242/dmm.050858

**Published:** 2024-10-01

**Authors:** Kondalarao Bankapalli, Ruth E. Thomas, Evelyn S. Vincow, Gillian Milstein, Laura V. Fisher, Leo J. Pallanck

**Affiliations:** Department of Genome Sciences, University of Washington, 3720 15th Avenue NE, Seattle, WA 98195, USA

**Keywords:** Tau, α-synuclein, Prion, Spread, Neurodegeneration, *Drosophila*

## Abstract

Brain protein aggregates are a hallmark of neurodegenerative disease. Previous work indicates that specific protein components of these aggregates are toxic, including tau (encoded by *MAPT*) in Alzheimer's disease and related tauopathies. Increasing evidence also indicates that these toxic proteins traffic between cells in a prion-like fashion, thereby spreading pathology from one brain region to another. However, the mechanisms involved in trafficking are poorly understood. We therefore developed a transgenic *Drosophila* model to facilitate rapid evaluation of candidate tau trafficking modifiers. Our model uses the bipartite Q system to drive co-expression of tau and GFP in the fly eye. We found age-dependent spread of tau into the brain, represented by detection of tau, but not of GFP. We also found that tau trafficking was attenuated upon inhibition of the endocytic factor dynamin (encoded by *shi*) or knockdown of glycogen synthase kinase-3β (GSK-3β, encoded by *sgg*). Further work revealed that dynamin promoted tau uptake in recipient tissues, whereas GSK-3β appeared to promote tau spread via direct phosphorylation of tau. Our robust and flexible system will promote the identification of tau-trafficking components involved in the pathogenesis of neurodegenerative diseases.

## Teaser

**Summary:** Our bipartite transgenic *Drosophila* model allows the analysis of tau protein spread to the brain and identifies the underlying mechanisms of tau trafficking in neurodegenerative diseases.

## INTRODUCTION

The accumulation of brain protein aggregates is a hallmark of neurodegenerative disease (Sengupta and Kayed, 2022; Lieberman et al., 2019; Moda et al., 2023). Although the composition of these aggregates, their subcellular distribution and their precise anatomical locations vary, studies using genetic, molecular and cell biological approaches have established that specific protein components of these aggregates are toxic and play central roles in pathogenesis (Bras et al., 2020; Samudra et al., 2023; Cavallucci et al., 2012; Chung et al., 2018). In particular, the protein tau (encoded by *MAPT*) is an essential component of the neurofibrillary tangles that mark Alzheimer's disease (AD), and α-synuclein (encoded by *SNCA*) is the major component of Lewy bodies in Parkinson's disease (PD) (Goedert et al., 2024; Morris et al., 2024). Increasing evidence also suggests that neurodegenerative diseases characterized by protein aggregates share fundamental features with prion diseases (Stopschinski and Diamond, 2017). Prion diseases are defined by their infectious nature and the fact that prions can spread between tissues (Ma and Wang, 2014). Although there is little evidence that other neurodegenerative diseases are infectious, an accumulating body of work indicates that the aggregates that characterize AD, PD, Huntington's disease and many other neurodegenerative diseases can move between tissues and thereby promote the spread of neurodegeneration (Davis et al., 2018). Both neurofibrillary tangles and Lewy bodies appear to propagate along defined neuroanatomical pathways as neurodegeneration progresses (Braak and Braak, 1991; Saito et al., 2004; Braak et al., 2003). Autopsies of patients with PD who had received fetal brain grafts revealed Lewy bodies in the graft tissue, suggesting that Lewy body pathology had propagated from the surrounding tissue (Li et al., 2008). Furthermore, injecting aggregated forms of tau or α-synuclein into the mouse brain recruited normally folded forms of these proteins into aggregates, which were later detected in brain regions far from the sites of injection (Clavaguera et al., 2009; Iba et al., 2015; Luk et al., 2012; Masuda-Suzukake et al., 2013).

Although recent work has begun to decipher the mechanisms by which toxic proteins spread in neurodegenerative disease, our understanding of these processes is far from complete (Uemura et al., 2020). A major factor limiting progress is the lack of model systems that permit rapid analysis of candidate trafficking components. To address this matter, we created a transgenic *Drosophila* strain that expresses tau, a toxic protein that aggregates in AD and related disorders (Creekmore et al., 2024). Although a number of transgenic lines have been created to study tau toxicity in *Drosophila*, these lines and the methods that have been used to monitor toxic protein trafficking have several important limitations (Jackson et al., 2002; Wittmann et al., 2001; Fernius et al., 2017; Aqsa and Sarkar, 2021; Donnelly and Pearce, 2018; Babcock and Ganetzky, 2015; Donnelly et al., 2020). Our new transgenic line, coupled with a rapid and simple detection method to detect tau spread, remedies these limitations. Our transgene, expressed under the control of the Q system (Potter et al., 2010), consists of the coding sequences of human tau and GFP with an intervening T2A protein cleavage sequence (Chng et al., 2015). GFP, which is cleaved from tau during translation and does not spread, marks the expression sites of our constructs; the spread of tau can thus be detected as locations where tau is present but GFP is not.

We found that expressing our *tau-T2A-GFP* construct in the fly eye resulted in abundant tau and GFP expression in the eye as expected. Tau protein was detected in the brain in increasing abundance over time, but GFP was never detected outside the eye, indicating that tau but not GFP spread to the brain. We also found that genetic perturbations that reduced the expression of the kinase glycogen synthase kinase-3β (GSK-3β) or the activity of the endocytic factor dynamin both resulted in reduced tau trafficking. These effects were specific to tau; the same perturbations had no effect on the spread of α-synuclein, the toxic component of the Lewy body aggregates that characterize PD (Saramowicz et al., 2023). Targeted perturbations of GSK-3β and dynamin in tissue subsets further showed that GSK-3β promotes tau spread by hyperphosphorylating tau, whereas dynamin promotes tau spread by fostering the uptake of tau in recipient cells. Together, our findings indicate that our novel method for testing candidate tau spread modifiers will prove valuable in helping to decipher the mechanisms underlying the spread of tau, and possibly of other toxic proteins involved in neurodegenerative disease.

## RESULTS

### Generation and expression of a *tau-T2A-GFP* transgenic line

**Fig. 1.**
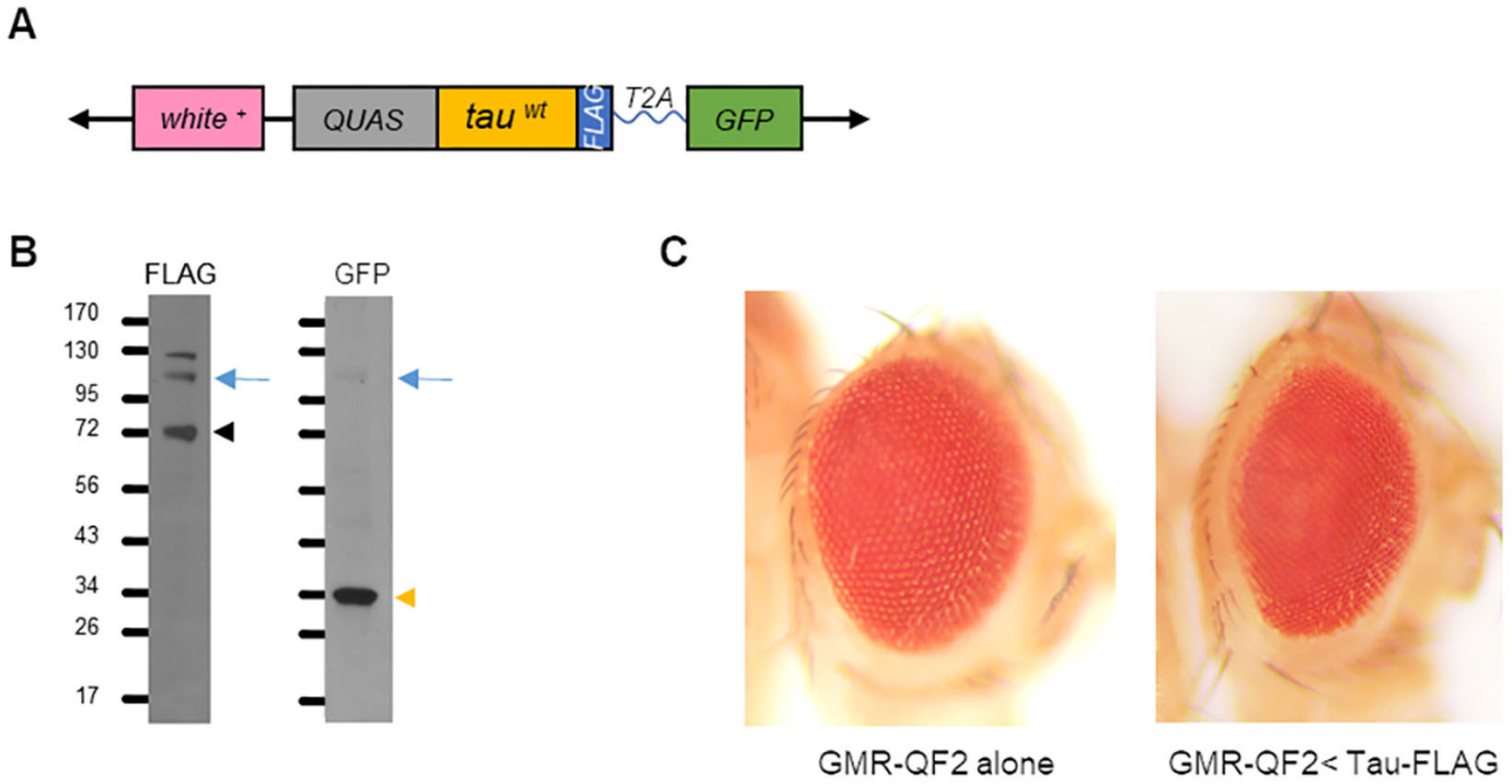
**Initial characterization of the *tau-T2A-GFP* transgenic line.** (A) Schematic description of the *tau-T2A-GFP* transgene. ‘*white^+^*’ represents the marker used to detect transgenic flies. ‘*QUAS*’ represents the Q-responsive regulatory sequence. The human tau coding sequence is flanked by a C-terminal FLAG tag coding sequence. (B) Western blot analysis of protein extracts from the heads of flies bearing the *tau-T2A-GFP* transgene and the eye-specific *GMR-QF2w* driver. Anti-FLAG antiserum (left lane) detected a tau-FLAG band of the expected size (∼70 kDa), indicated by the black arrowhead, and anti-GFP antiserum detected a GFP band of the expected size (∼30 kDa), indicated by the yellow arrowhead. Fainter bands of ∼100 kDa were also detected by both anti-FLAG and anti-GFP antisera at the expected size of the *tau-T2A-GFP* fusion protein (blue arrows), indicating that T2A cleavage is efficient but not complete. *n*≥3 experiments. (C) Expression of the *tau-T2A-GFP* construct in the eye using the *GMR-QF2w* driver resulted in an eye that was small and mildly rough compared to the eyes of flies bearing only the driver.

The goal of our work was to create a model system suitable for rapid screening of candidate factors that influence tau trafficking between tissues. Our system involves three features. First, we used the Q system (Potter et al., 2010) to drive tau expression such that we could independently use the GAL4 system (Brand and Perrimon, 1993) to perturb candidate trafficking components. The Q system works similarly to the GAL4 system in that the tissue-specific expression of the exogenous transcription factor QF2 selectively drives the transcription of transgenes that contain a QF2 transcriptional response element. Importantly, QF2 does not recognize the GAL4 transcriptional response element and, therefore, does not interfere with the GAL4 expression system transgenes (Potter et al., 2010; Lin and Potter, 2016). Second, to visualize tau spread while simultaneously marking the original site of expression, we generated a QF2-responsive construct consisting of the coding sequence of human tau tagged with FLAG, followed by a T2A protein self-cleavage sequence (Chng et al., 2015), and then by the coding sequence of GFP (Fig. 1A). The T2A sequence releases GFP from tau, such that GFP marks the tissues where our transgene is expressed, whereas tissues that express tau independently of GFP represent tau spread. Third, to facilitate rapid screening, we developed a simple western blot procedure to detect tau spread.

After generating transgenic lines with the *tau-T2A-GFP* transgene, we crossed them to a stock bearing the eye-specific *GMR-QF2w* driver. Head protein extracts from the resulting offspring were then tested for transgene expression by western blotting for FLAG and GFP. The constructs produced appropriately sized tau-FLAG and GFP proteins. The expected cleavage of GFP from tau appeared to be efficient, with only a minor amount of tau-GFP fusion protein detected (Fig. 1B). We also tested the effects of transgene expression using a known bioassay: disruption of the fly eye. Tau expression is toxic in many tissues and has been shown to produce a ‘rough eye’ phenotype when expressed in the eye (Jackson et al., 2002; Wittmann et al., 2001). Consistent with these published findings, we found that eye-specific expression of the *tau-T2A-GFP* construct caused a mild rough eye phenotype (Fig. 1C).

### Analysis of tau spread

**Fig. 2.**
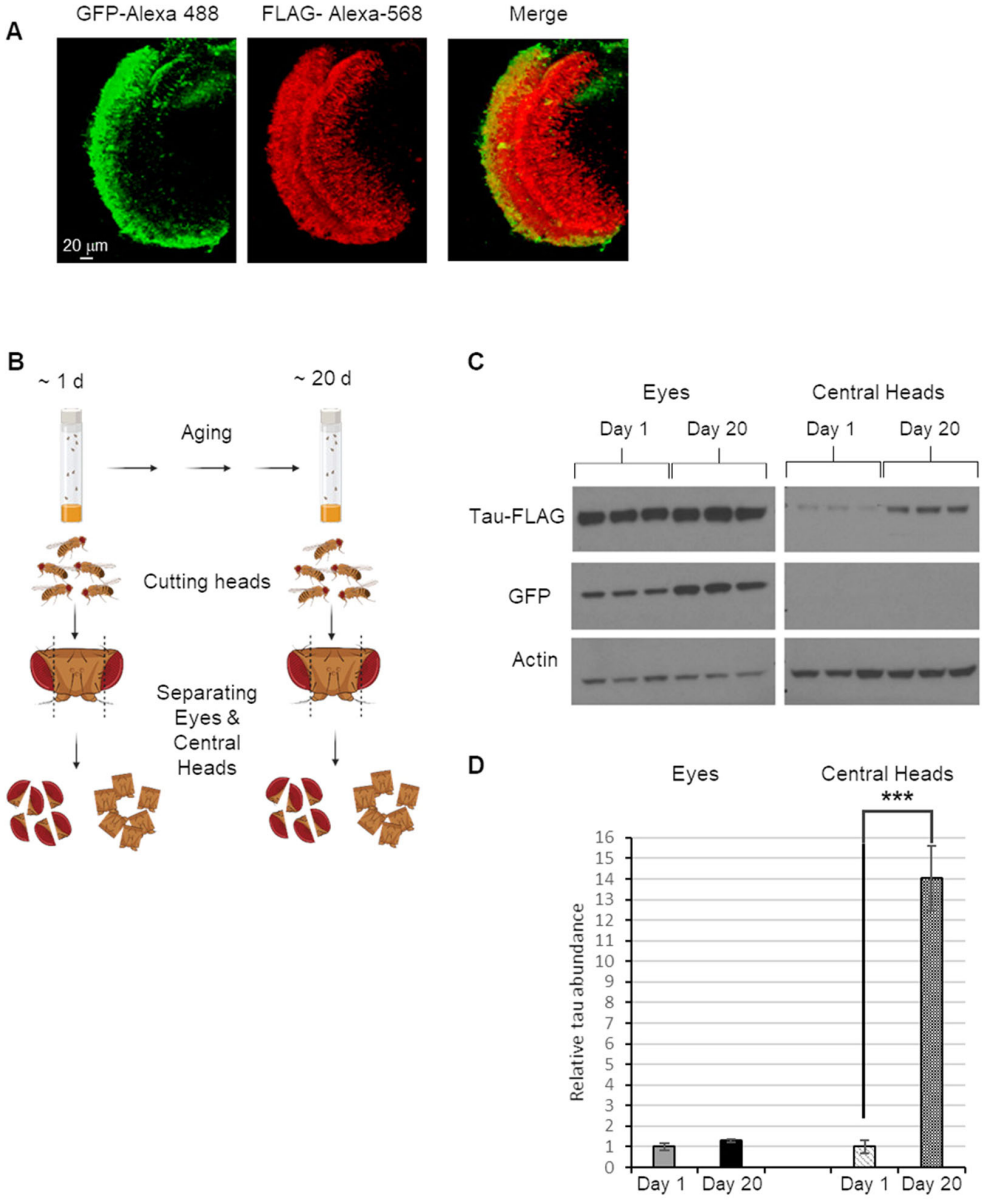
**Human tau expressed in the fly eye is detected in the brain and accumulates with age.** (A) Confocal images of eye and optic lobe from the brains of 40-day-old flies expressing the *tau-T2A-GFP* construct under control of the eye-specific *GMR-QF2w* driver. Tau expression and spread were detected using anti-GFP (green) and anti-FLAG (red) antibodies, respectively. *n*≥3 experiments. (B) Schematic of the experimental approach to measure tau spread. (C) Western blot analysis using antisera against FLAG, GFP and actin, performed on protein extracts from the eyes and central heads of 1- and 20-day-old flies expressing tau in the eye. As in panel A, the flies bore both the *GMR-QF2w* driver and the *tau-T2A-GFP* construct. Each condition was represented by three biological replicates. (D) Quantification of tau abundance normalized to actin levels using the data shown in panel C. Data are mean±s.e.m. ***P<0.005 by unpaired two-tailed Student's *t-*test.

Previous work in *Drosophila* has shown that targeted expression of human tau protein leads to progressive spread of tau to other brain regions (Aqsa and Sarkar, 2021). To test whether tau expression using our constructs also led to spread beyond the site of expression, we used two approaches. First, we dissected the brains from animals expressing tau using the *GMR-QF2w* driver and performed immunocytochemistry and confocal microscopy using antisera against GFP and tau. This analysis revealed abundant tau and GFP expression in the retina as expected (Fig. 2A). Tau protein was also detected in other brain regions in the absence of GFP, indicating that tau had spread from the retina to the optic lobe (Fig. 2A). These results confirmed previously published work demonstrating that tau spreads from the fly eye to other brain regions (Aqsa and Sarkar, 2021). However, the methods used in previous work to detect tau spread are not suitable for rapid screening of candidate trafficking modifiers. In particular, it is difficult to quantify spread accurately using immunocytochemistry, and the process of brain dissection, immunocytochemical staining and confocal microscopy is laborious and time-consuming. We therefore developed a novel method for detecting tau spread that was simple and rapid. We separated fly eyes from heads using a razor blade (Fig. 2B) and created separate extracts from the dissected eyes and the heads without eyes (henceforth ‘central heads’). Immunoblotting of these extracts revealed abundant tau and GFP protein expression in the eyes, as expected for the original site of expression of the constructs (Fig. 2C). Central head extracts, in contrast, contained tau protein but never GFP. This finding confirmed that our method was capable of detecting the spread of tau protein beyond the area in which it was expressed. The abundance of tau detected in central heads increased as flies aged, mimicking the age-dependent spread of toxic proteins in neurodegenerative disease (Fig. 2C,D).

### Exploring the influence of candidate modifiers of tau spread

**Fig. 3.**
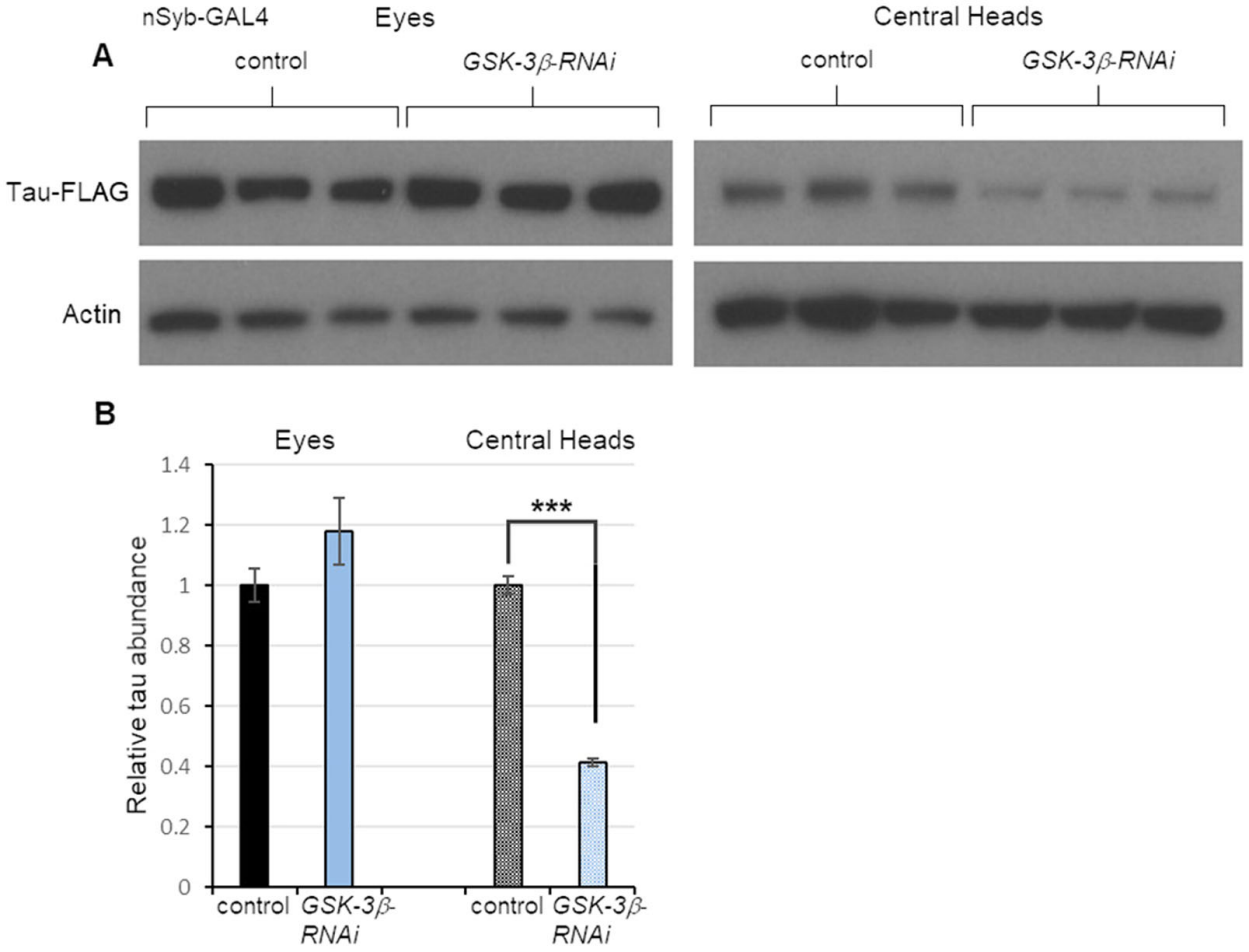
**Knockdown of *GSK-3β* decreases tau spread.** (A) Western blot analysis of protein extracts from the eyes and central heads of 20-day-old flies expressing tau in the eye and RNAi to *GSK-3β* in neurons. Specifically, *GMR-QF2w* drove *tau-T2A-GFP* expression, and the pan-neuronal driver *nSyb-GAL4* drove expression of a GAL4-responsive RNAi transgene targeting *GSK-3β*. Control flies lacked the RNAi transgene. Each condition was represented by three biological replicates. (B) Quantification of tau abundance normalized to actin levels using the data shown in panel A. Data are mean±s.e.m. ***P<0.005 by unpaired two-tailed Student's *t-*test.

Having validated our system, we tested whether it could be used to identify modifiers of tau spread. One of the candidate factors we tested was GSK-3β. Previous work has established that hyperphosphorylation of tau contributes to its toxicity (Drewes, 2004) and that GSK-3β is among the kinases responsible for this hyperphosphorylation (Rankin et al., 2007). GSK-3β is also a modifier of tau toxicity in *Drosophila* and a modifier of PD risk (Jackson et al., 2002; Sayas and Avila, 2021; Li et al., 2014). Most importantly, recent work with another *Drosophila* model of tau spread has shown that *GSK-3β* knockdown decreases tau spread as measured by immunohistochemistry (Aqsa and Sarkar, 2021). To independently validate the role of GSK-3β in tau spread, we performed knockdown of this factor in flies expressing the *tau-T2A-GFP* transgene. Specifically, we used the pan-neuronal driver *nSyb-GAL4* to express RNAi targeting *GSK-3β*. We found that *GSK-3β* knockdown significantly reduced tau spread from the eye to the brain (Fig. 3A,B). Importantly, tau abundance in the eye was not affected, indicating that the reduced abundance of tau in central heads resulted from reduced spread rather than reduced expression. These results support previous findings that GSK-3β influences tau spread and they establish the feasibility of our simple and rapid approach for testing candidate trafficking factors.

**Fig. 4.**
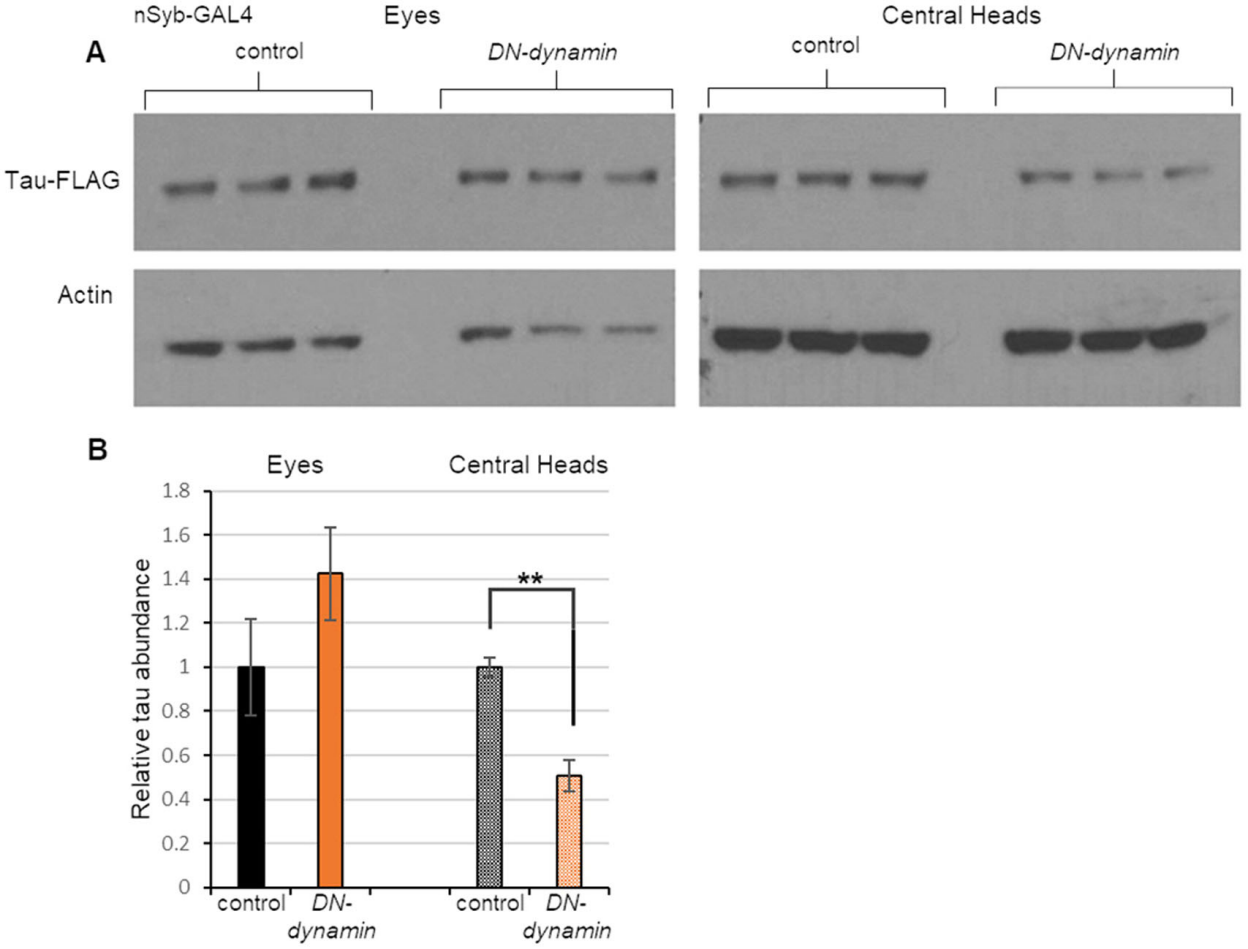
**Genetic inhibition of *dynamin* decreases tau spread.** (A) Western blot analysis of protein extracts from the eyes and central heads of 15-day-old flies expressing tau in the eye and a dominant-negative (DN) form of dynamin in neurons. The experimental group bore *GMR-QF2w* driving *tau-T2A-GFP* expression and *nSyb-GAL4* driving DN-dynamin expression. Control flies lacked the GAL4-responsive transgene. Each condition was represented by three biological replicates. (B) Quantification of tau abundance normalized to actin levels using the data shown in panel A. Data are mean±s.e.m. **P<0.01 by unpaired two-tailed Student's *t-*test.

The next candidate factor we chose to interrogate was the endocytosis factor dynamin. Work in both vertebrate and invertebrate model systems strongly suggests that endocytosis promotes the spread of toxic proteins involved in neurodegeneration by facilitating uptake of the protein in recipient cells (Uemura et al., 2020; Babcock and Ganetzky, 2015). We therefore tested whether inactivating dynamin in flies co-expressing the *tau-T2A-GFP* transgene in the eye would reduce the spread of tau. To perform this experiment, we used *nSyb-GAL4* to express a dominant-negative form of dynamin (Kitamoto, 2001) in neurons. We found that expression of the dominant-negative *dynamin* transgene significantly reduced tau spread from the eye to the brain. As was the case with *GSK-3β* knockdown, tau abundance in the eye was not detectably affected by expression of dominant-negative dynamin, again indicating that the reduction of tau spread to the brain was not a secondary consequence of reduced tau expression (Fig. 4A,B).

**Fig. 5.**
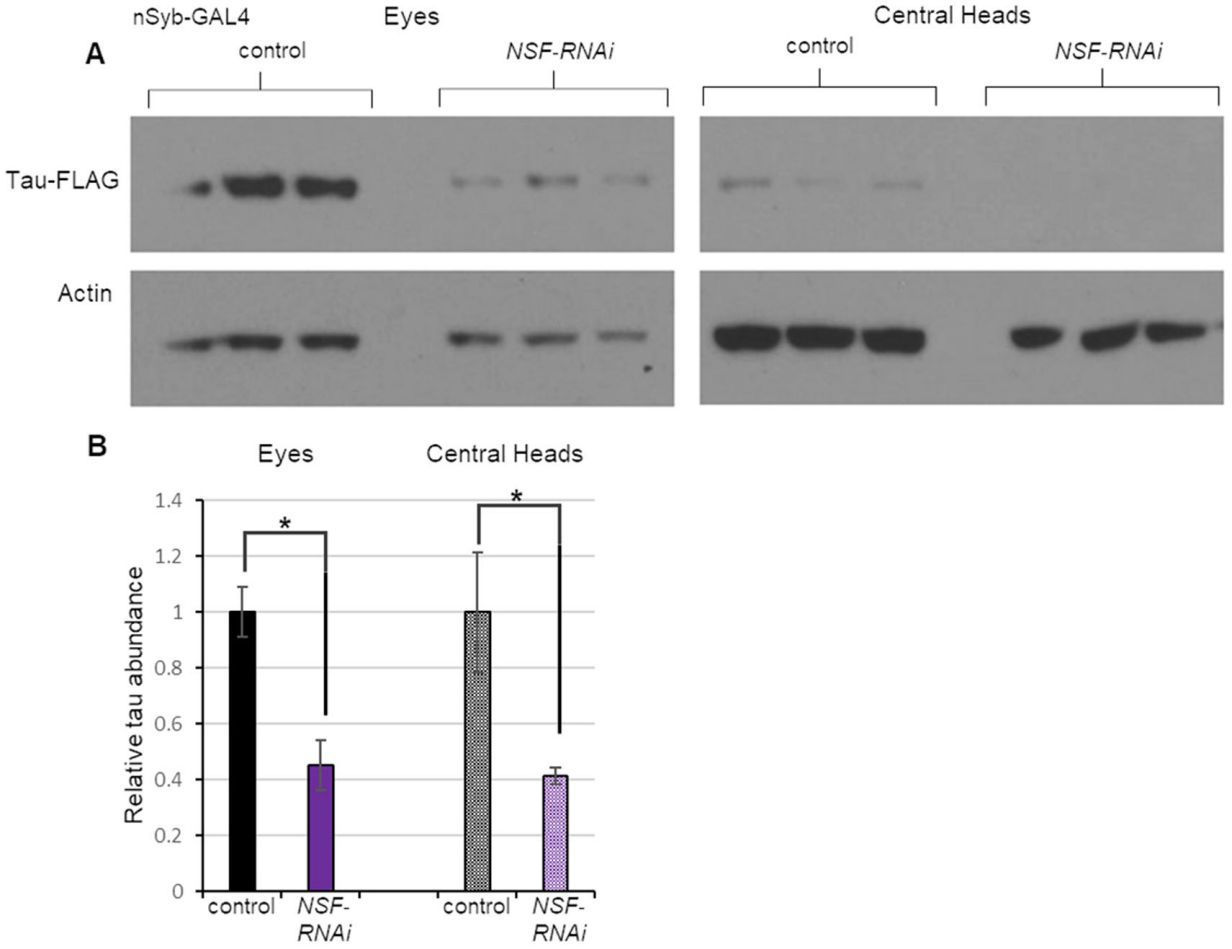
**Tau abundance and spread are equally reduced upon knockdown of *NSF*.** (A) Western blot analysis of protein extracts from the eyes and central heads of 30-day-old flies expressing *tau-T2A-GFP* in the eye and RNAi targeting *NSF* in neurons. The experimental group bore *GMR-QF2w* driving *tau-T2A-GFP* expression and *nSyb-GAL4* driving expression of a GAL4-responsive RNAi transgene to *NSF*. Control flies lacked the RNAi transgene. Each condition was represented by three biological replicates. (B) Quantification of tau abundance normalized to actin levels using the data shown in panel A. Data are mean±s.e.m. *P<0.05 by unpaired two-tailed Student's *t-*test.

Work in model systems has suggested that tau protein spread occurs at neuronal synapses (Robbins et al., 2021). Moreover, a *Drosophila* study previously showed that knockdown of the *N-ethylmaleimide-sensitive factor, vesicle-fusing ATPase* (*NSF*) gene (also known as *comatose* or *comt*), which plays a critical role in synaptic vesicle recycling, reduced the spread of the huntingtin (Htt) protein (Babcock and Ganetzky, 2015). Thus, we tested whether using the pan-neuronal *nSyb-GAL4* driver to express RNAi against *Drosophila NSF* would reduce the spread of human tau from the fly eye. The amount of tau detected in central heads was greatly reduced upon knockdown of *NSF* (Fig. 5A,B). However, the overall abundance of tau in the eye was also severely reduced by this manipulation (Fig. 5A,B) and the extent of tau reduction was similar in the two locations. Taken together, these findings suggest that the reduced spread of tau seen upon *NSF* knockdown is likely a secondary consequence of reduced tau expression rather than altered spread. Although this finding was unexpected, it reveals a useful feature of our system: it facilitates the distinction between genetic factors that influence tau spread and those that merely appear to affect tau spread as a consequence of their effect on tau abundance.

### Testing whether tau spread modifiers act cell-autonomously or non-cell-autonomously

**Fig. 6.**
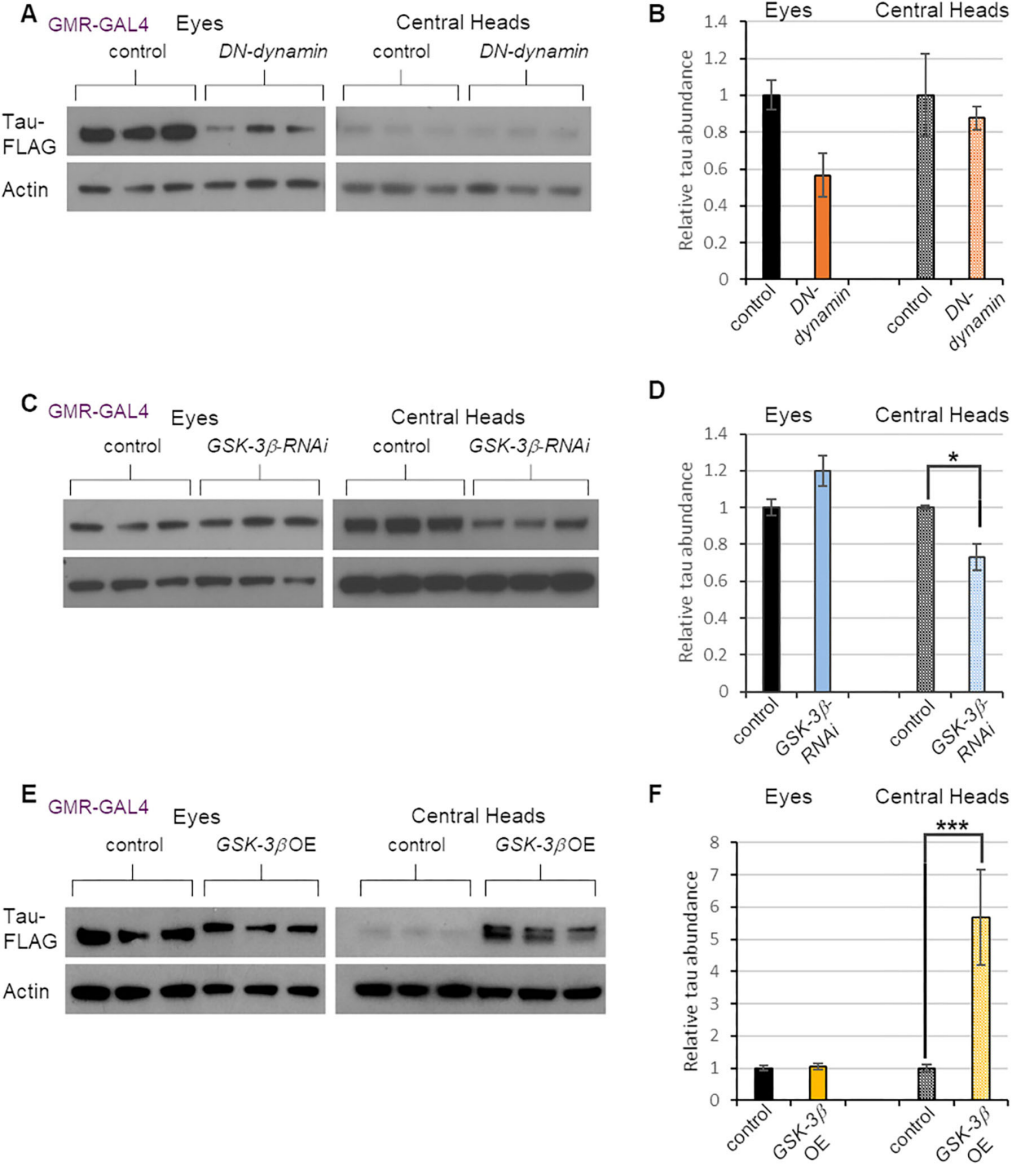
**Dynamin influences tau spread in recipient tissues, whereas GSK-3β affects tau spread in source tissues.** (A) Western blot analysis of protein extracts from the eyes and central heads of 20-day-old flies expressing both tau and dominant-negative (DN) dynamin in the eye. The experimental groups bore *GMR-QF2w* driving *tau-T2A-GFP* expression and *GMR-GAL4* driving expression of a GAL4-responsive DN-dynamin construct. Control flies lacked the transgene encoding DN-dynamin. (B) Quantification of tau abundance normalized to actin levels using the data in panel A. (C) Western blot analysis of protein extracts from the eyes and central heads of 20-day-old flies expressing both tau and RNAi to *GSK-3β* in the eye. The experimental groups bore *GMR-QF2w* driving *tau-T2A-GFP* expression and *GMR-GAL4* driving expression of a GAL4-responsive RNAi transgene to *GSK-3β.* Control flies lacked the *GSK-3β* RNAi transgene. (D) Quantification of the data in panel C. (E) Western blot analysis of protein extracts from the eyes and central heads of 20-day-old flies expressing tau and overexpressing (OE) *GSK-3β* in the eye. The experimental groups bore *GMR-QF2w* driving *tau-T2A-GFP* expression and *GMR-GAL4* driving expression of a GAL4-responsive *GSK-3β* construct. Control flies lacked the *GSK-3β* transgene. (F) Quantification of the data in panel E. For all experiments described in this figure, each condition was represented by three biological replicates. Data are mean±s.e.m. *P<0.05; ***P<0.005 by unpaired two-tailed Student's *t-*test.

We next sought to determine whether factors influencing tau spread acted in the cells or tissues where tau was originally expressed (source tissues), or in the cells or tissues to which tau spread (recipient tissues). To distinguish between these possibilities, we repeated our genetic perturbations, this time using the *GMR-GAL4* driver to target our perturbations to the eye. The dominant-negative *dynamin* construct, which reduced tau spread when driven in neurons, had no effect on tau spread when driven in the eye (Fig. 6A,B). However, eye-specific expression of the *GSK-3β* RNAi caused a decrease in tau spread (Fig. 6C,D) that was comparable to the decrease seen with *nSyb-GAL4* (Fig. 3). To further explore the influence of GSK-3β on tau spread, we used the *GMR-GAL4* driver to express a GAL4-responsive *GSK-3β* transgene in the eyes of tau-expressing flies. This manipulation resulted in a dramatic increase in tau spread (>5-fold relative to controls; Fig. 6E,F). Overexpressing GSK-3β also caused a mobility shift in tau on western blot that was consistent with phosphorylation, in accordance with previous work showing that tau is a direct substrate of GSK-3β (Rankin et al., 2007). Taken together, these findings indicate that the endocytic factor dynamin promotes tau uptake in recipient tissues, whereas GSK-3β­­ activity promotes tau spread by hyperphosphorylating tau in source tissues.

### Exploring the specificity of the tau spread modifiers

**Fig. 7.**
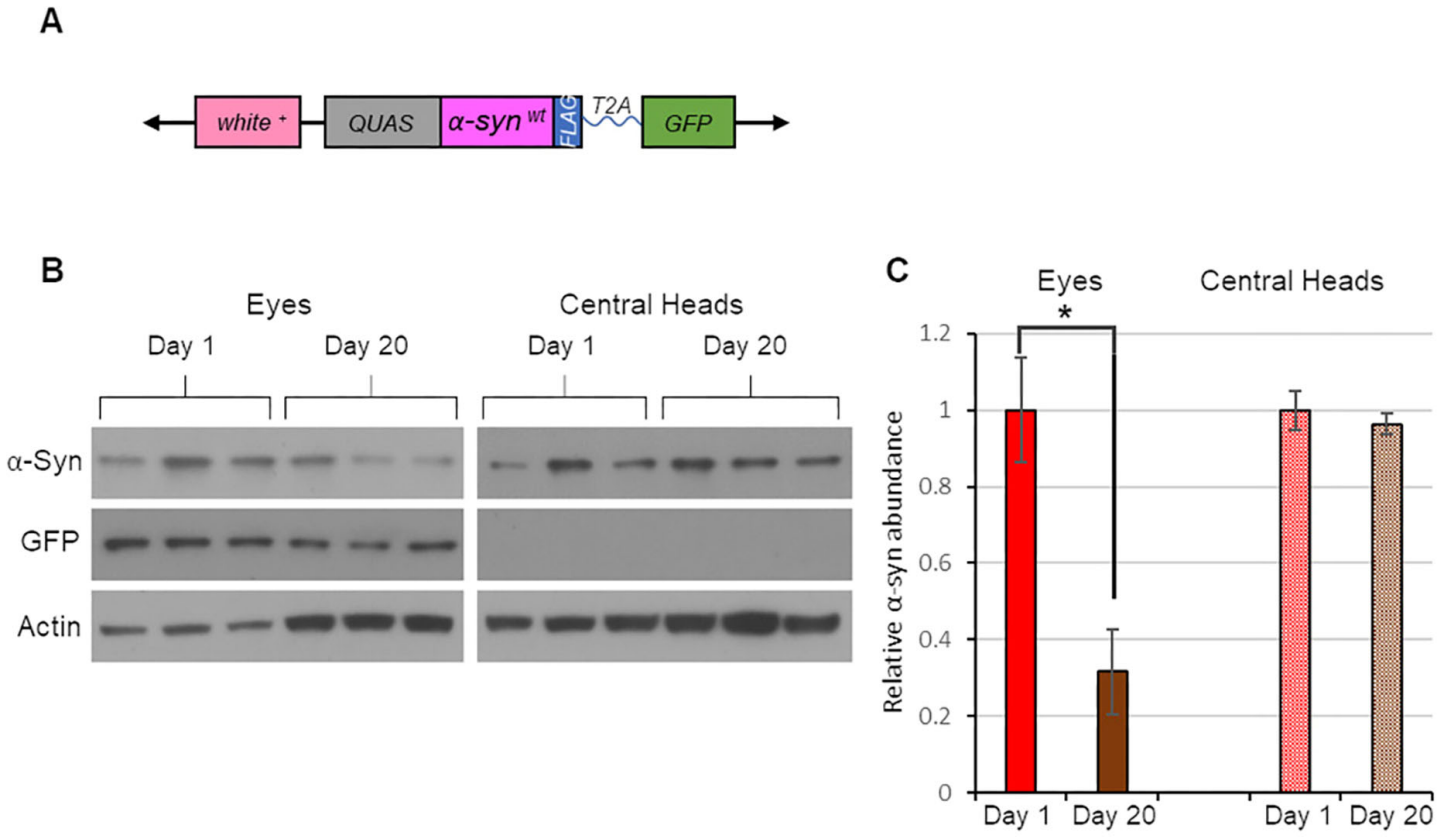
**α-synuclein expressed in the fly eye spreads to the brain, but spread is not progressive with age.** (A) Schematic description of the *α-synuclein-T2A-GFP* transgene (see Fig. 1A). (B) Western blot analysis using antisera against α-synuclein, GFP and actin, performed on protein extracts from the eyes and central heads of 1- and 20-day-old flies expressing α-synuclein in the eye. Flies bore the *GMR-QF2w* driver and the *α-synuclein-T2A-GFP* transgene. Each condition was represented by three biological replicates. (C) Quantification of α-synuclein abundance using the data in panel B. Data are mean±s.e.m. *P<0.05 by unpaired two-tailed Student's *t*-test.

Many neurodegenerative disorders are characterized by brain protein aggregates that appear to spread between brain regions. This raises an important question: are the mechanisms of toxic protein spread shared among neurodegenerative diseases or are they disease specific? To begin to address this matter, we created a transgenic line that expresses the α-synuclein protein, a major component of the Lewy body brain protein aggregates that are observed in PD and related disorders (Saramowicz et al., 2023; Henderson et al., 2019). Substantial evidence indicates that excess α-synuclein is toxic and that it has the ability to spread between brain regions (Uemura et al., 2020; Henderson et al., 2019; Nussbaum, 2018). Our α-synuclein transgenic line was created in the same fashion as our tau transgenic line. Specifically, we created a Q-responsive transgenic construct that contained the coding sequences of α-synuclein and GFP with an intervening T2A cleavage peptide (Fig. 7A). Driving this transgene in the eye with *GMR-QF2w* produced the expected expression of α-synuclein and GFP (Fig. 7B). α-synuclein was also detected in central heads but GFP was not, indicating that α-synuclein spreads beyond its site of expression as tau does (Fig. 7B). However, unlike tau, the abundance of α-synuclein in central heads did not increase with adult age (Fig. 7C), suggesting that maximum spread of α-synuclein occurs during the pupal stage of development.

**Fig. 8.**
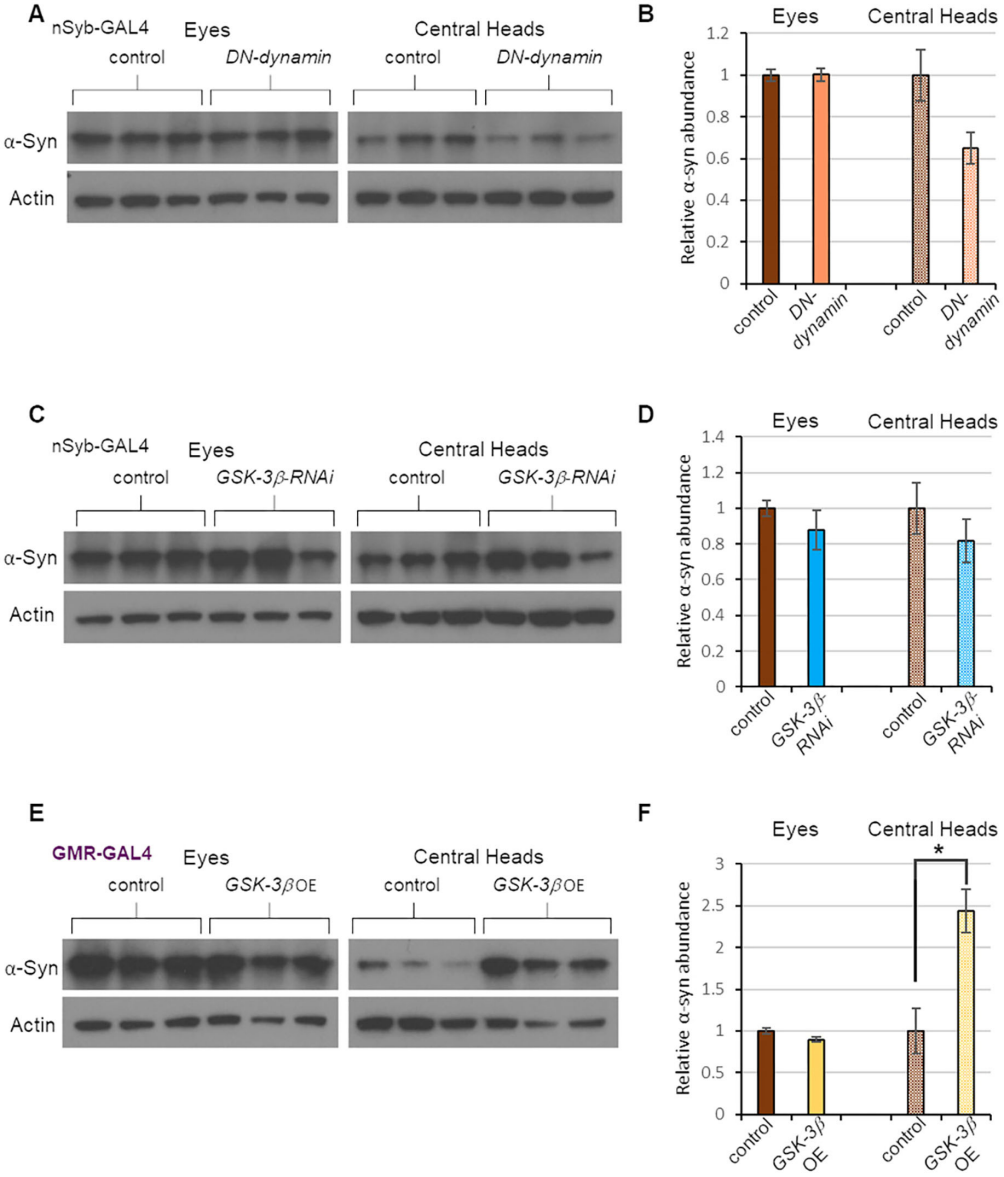
**Tau spread modifiers have little or no influence on α-synuclein spread.** (A) Western blot analysis of protein extracts from the eyes and central heads of 20-day-old flies expressing α-synuclein in the eye and a dominant-negative (DN) form of dynamin in neurons. The experimental group bore *GMR-QF2w* driving *α-synuclein-T2A-GFP* expression and *nSyb-GAL4* driving expression of a dominant-negative (DN) dynamin construct. Control flies lacked the transgene encoding the DN form of dynamin. (B) Quantification of the data in panel A. (C) Western blot analysis of protein extracts from the eyes and central heads of 20-day-old flies expressing α-synuclein in the eye and RNAi to *GSK-3β* in neurons. Specifically, *GMR-QF2w* drove *α-synuclein-T2A-GFP* expression, and the pan-neuronal driver *nSyb-GAL4* drove expression of a GAL4-responsive RNAi transgene targeting *GSK-3β*. Control flies lacked the *GSK-3β* RNAi transgene. (D) Quantification of the data in panel C. (E) Western blot analysis of protein extracts from the eyes and central heads of 20-day-old flies expressing *α-syn-T2A-GFP* in the eye using *GMR-QF2w* and co-expressing a GAL4-responsive *GSK-3β* transgene using *GMR-GAL4*. Control flies lacked the *GSK-3β* transgene. OE, overexpression. (F) Quantification of the data in panel E. For all experiments described in this figure, each condition was represented by three biological replicates. Data are mean±s.e.m. *P<0.05 by unpaired two-tailed Student's *t*-test.

We then tested whether factors that influenced tau spread also influenced α-synuclein spread. Specifically, we repeated our experiments with the RNAi targeting *GSK-3β* and the dominant-negative *dynamin* construct in flies expressing our *α-synuclein-T2A-GFP* construct. These perturbations had no significant effect on the spread of α-synuclein (Fig. 8A-D). When we overexpressed GSK-3β, we found an increase in α-synuclein spread (Fig. 8E,F), but the effect of this manipulation on α-synuclein was considerably smaller than its previous effect on tau (Fig. 3). Overall, our findings indicate that most of the genetic perturbations that influence tau spread were specific to tau.

## DISCUSSION

The prion-like spread of toxic proteins from one brain region to another has gained increasing acceptance as an important pathological feature of many neurodegenerative diseases (Ma et al., 2019; Ayers et al., 2018; Ayers and Cashman, 2018; Pearce and Kopito, 2018). Over the past several years, this process has been recapitulated in a number of model systems, including *Drosophila* (Aqsa and Sarkar, 2021; Babcock and Ganetzky, 2015; Donnelly et al., 2020). However, this recent work has several limitations. First, it can be challenging to distinguish between spread of the toxic protein and mild misexpression of the gene encoding the toxic protein outside of the designated target tissues. Second, vertebrate systems are not suitable for rapid exploration of candidate pathways because of the time involved in generating and aging the animals required. Third, tissue dissection and confocal microscopy to assess the extent of toxic protein spread is both time consuming and not readily amenable to quantification, particularly in instances where the differences between genotypes are modest. Our approach overcomes all of these limitations by creating transgenic *Drosophila* expressing tau and GFP from the same transgene and using a simple western blot procedure to detect the migration of tau. Moreover, because our system makes use of the Q expression system to drive tau expression, the many existing GAL4-responsive reagents can be used to knock down and overexpress candidate spread modifiers in virtually any desired tissue without influencing tau expression.

Our work is supported by previous evidence that *Drosophila* can be used to study the mechanisms underlying the spread of neurodegeneration-associated proteins (Aqsa and Sarkar, 2021; Babcock and Ganetzky, 2015; Donnelly et al., 2020). We now provide a set of tools for systematic study of this phenomenon. Importantly, several of our observations indicate that the spread of tau in *Drosophila* reflects the processes involved in the spread of tau in the human brain. First, the spread of tau does not appear to be a secondary consequence of loss of cellular integrity due to tau toxicity; if this were the case, GFP would also spread. Second, the fact that GFP does not spread is not due to the smaller size of GFP compared to that of tau. Our *α-synuclein-T2A-GFP* flies also showed spread of their disease-associated protein, and α-synuclein is not larger but smaller than GFP. Third, age-dependent differences in the spread of tau and α-synuclein oppose the idea that spread could be a common artifact of our model system and imply that tau and α-synuclein spread through distinct mechanisms. Further support for the idea of distinct spread mechanisms comes from the finding that most of the genetic perturbations that affected tau spread in our experiments did not detectably affect α-synuclein spread. Finally, our findings on specific candidate spread markers are consistent with work in other model systems, including vertebrates.

Our work also points to potential mechanisms underlying the spread of tau protein. Specifically, we found that genetic perturbations that reduced or eliminated the activity of *Drosophila* dynamin or GSK-3β resulted in reduced tau spread. Both findings are consistent with previous work in *Drosophila* and mammals (Aqsa and Sarkar, 2021; Babcock and Ganetzky, 2015; Amaral et al., 2021; Wu et al., 2013). For example, a *Drosophila* model of huntingtin protein toxicity demonstrated that selectively blocking endocytosis, using the same dominant-negative *dynamin* construct used in our work, prevented non-cell-autonomous huntingtin-mediated degeneration (Babcock and Ganetzky, 2015). The influence of dynamin on huntingtin protein toxicity was seen in the tissues that acquired huntingtin protein from surrounding tissues (Babcock and Ganetzky, 2015), consistent with our observations showing that pan-neuronal inactivation of dynamin reduced tau spread, but selective inhibition of dynamin in the eye did not. Of even greater relevance to our current work, a recent *Drosophila* study showed that *GSK-3β* knockdown reduced tau spread from the eye to the brain (Aqsa and Sarkar, 2021). However, although the previous work used the GAL4 system to express both tau and the RNAi targeting *GSK-3β*, our system allowed us to manipulate GSK-3β expression independently of tau. We were therefore able to show that GSK-3β is selectively required in the donor tissue to influence tau spread. Furthermore, although we did not directly test phosphorylation levels, our findings suggested that the effect of overexpressing GSK-3β was at least partly mediated by hyperphosphorylation of tau. Previous work has shown that tau is a direct substrate of GSK-3β (Rankin et al., 2007) and that phosphorylation increases tau toxicity (Drewes, 2004). Our experiments showed that overexpression of GSK-3β caused a mobility shift for tau protein that was consistent with increased phosphorylation. Taken together, these findings strongly imply that the influence of GSK-3β on tau spread is at least partially a consequence of tau hyperphosphorylation. The mechanism by which tau hyperphosphorylation would influence its spread is unclear from our work, but one previously proposed explanation is that the reduced affinity of phosphorylated tau for microtubules might trigger its misfolding and secretion through a non-canonical secretory mechanism (Zhang et al., 2021).

Previous work has also shown that GSK-3β can phosphorylate α-synuclein on serine 129 (Credle et al., 2015) and that serine 129 phosphorylation increases α-synuclein toxicity in multiple model systems (Chen and Feany, 2005; Sato et al., 2011; Kragh et al., 2009). Our finding that GSK-3β overexpression increases α-synuclein spread raises the possibility that α-synuclein is more toxic upon phosphorylation of serine 129 because this phosphorylation event alters α-synuclein spread. However, as GSK-3β overexpression had no obvious influence on α-synuclein mobility in our western blot assays, our findings are also consistent with the possibility that the influence of GSK-3β on α-synuclein spread is indirect. Although GSK-3β overexpression does affect tau mobility, indicating that tau is likely a direct target GSK-3β activity, the influence of GSK-3β on tau spread may also be at least partially indirect. Our new model system will facilitate efforts to resolve these and other questions.

A strength of our approach is its ability to detect and rule out candidate spread modifiers that alter overall tau abundance. For instance, we found that *NSF* knockdown caused a reduction of tau abundance in the brain, consistent with an earlier report that the same manipulation reduced huntingtin spread in *Drosophila* (Babcock and Ganetzky, 2015). If not for the simplicity and sensitivity of detecting differences in tau abundance within the source tissue (eye) afforded by our methodology, we would have classified NSF as a modifier of tau spread rather than a modifier of tau abundance. Furthermore, our work on NSF revealed another useful feature of our system. We found that the effect of *NSF* knockdown on tau abundance was specific to tau, because GFP expressed from the same transgene was not reduced in abundance by this manipulation (Fig. S1). Taken together, these findings indicate that the effect of *NSF* knockdown on tau abundance is not an artifact of the expression systems used or a general defect in expression, but rather a specific effect of NSF on tau abundance. Although we cannot at present explain how reduced NSF expression selectively alters tau abundance, previous work has shown that NSF interacts physically (Zhao et al., 2007) and genetically (Peyre et al., 2006) with cytoskeletal components. Furthermore, tau itself has recently been shown to interact with NSF and reduce its activity (Prikas et al., 2022). Regardless of how NSF alters tau abundance, these results further demonstrate the utility and power of our model to identify and categorize tau modifiers.

Over the past 20 years, researchers have identified hundreds of genetic loci that influence the risk of AD, PD and other neurodegenerative disorders associated with the accumulation and spread of protein aggregates in the brain (Guo et al., 2022; Kaivola et al., 2023; Andrade-Guerrero et al., 2023; Fang et al., 2022; Lake et al., 2023; Guerreiro et al., 2020; Fernandez-Santiago and Sharma, 2022; Grenn et al., 2020). However, for many of these loci, we do not know how they influence disease risk, or precisely which gene in the linkage region is the risk-modifying factor. Although these loci likely influence risk in a variety of ways, we anticipate that at least some act by enhancing the spread of tau or α-synuclein to other brain regions. Thus, we anticipate that our model systems for identifying modifiers of tau and α-synuclein spread will be extremely useful in identifying genes that influence the risk of AD and PD and the mechanisms by which they act. Knowledge acquired from these studies could ultimately lead to the development of therapeutic strategies designed to block the spread of tau and α-synuclein, and thus to prevent or slow development of neurodegenerative disease.

## MATERIALS AND METHODS

### Fly stocks and generation of transgenic lines

All fly stocks were maintained on regular cornmeal-molasses food at 25°C using a 12-h/12-h light/dark cycle. The *GMR-QF2w* (#59283), *nSyb-GAL4* (#51941) and *GMR-GAL4* (#8121) driver stocks were obtained from the Bloomington *Drosophila* Stock Center (BDSC). Stocks bearing RNAi constructs targeting potential spread modifiers were also acquired from the BDSC: *UAS-sgg-RNAi* (#31309) and *UAS-sgg* (#5361) for RNAi against and overexpression of *GSK-3β*, respectively; *UAS-shi^ts^* (#44222; *dynamin*); and *UAS-comatose-RNAi* (#31666; *NSF*).

To produce the transgenic fly lines created for this study, we first generated two recombinant constructs in the pQUAS_WALIUM20 plasmid (#1474, *Drosophila* Genomics Resource Center), one with the wild-type (2N4R) human tau sequence and the other with wild-type α–synuclein sequence (see Fig. 1A) with the assistance of Blue Heron Biotech. Both disease-associated protein-coding sequences were thus placed under *QUAS* promoter control. The sequence of tau or α-synuclein was followed by a FLAG sequence, a cleavable T2A domain, and then the coding sequence of GFP. These constructs were then injected into *Drosophila* embryos to generate transgenic flies with the assistance of Rainbow Transgenic Flies, Inc.

### Immunohistochemistry

Brains from 40-day-old female flies were dissected in ice-cold 1× phosphate-buffered saline (PBS, pH 7.5) and fixed in 4% paraformaldehyde in PBS for 45 min. Tissues were washed in 1× PBS containing 0.1% Triton X-100. Fixed brains were probed with 1:500 rabbit anti-FLAG (Cell Signaling Technology, 14793S) and 1:800 mouse anti-GFP (Biolegend, 668205) primary antibodies overnight at 4°C, followed by staining with Alexa Fluor 488-conjugated anti-mouse IgG (Invitrogen, A-11001) and Alexa Fluor 568-conjugated anti-rabbit IgG (Invitrogen, A-11036) antibodies. Samples were mounted using ProLong Gold Antifade (Molecular Probes, P10144), and imaging was performed using an SP8 confocal microscope (Leica).

### Dissections and western blotting

Groups of 18 to 20 female flies were harvested by flash freezing at appropriate time points, most at day 20 or 21, with age-matched controls. Ages are indicated in the legend of each figure. Under a dissecting microscope, eyes were separated from central heads using a razor blade. Sets of eyes and sets of central heads were homogenized separately, each in 100 µl of 1× RIPA lysis buffer (50 mM Tris-HCl, pH 7.4; 150 mM NaCl; 1% Nonidet P-40; 0.5% sodium deoxycholate; 0.1% SDS). Proteins were separated by SDS-PAGE using 4-20% MOPS-acrylamide gels (GenScript Express Plus, M42012) and transferred electrophoretically onto Immobilon PVDF membrane (Merck). The membrane was transferred to blocking buffer (5% non-fat dry milk in 1× PBS with 0.1% Tween 20) for 1 h. Membranes were incubated overnight with primary antibodies diluted in blocking buffer. Primary antibody dilutions were as follows: 1:1000 rabbit anti-FLAG (Cell Signaling Technology, 14793S), 1:1000 anti-α-synuclein (BD Transduction Laboratories, 610787), 1:500 mouse anti-GFP (Biolegend, 668205) and 1:5000 mouse anti-β-actin (Millipore Sigma, MAB1501). After three washes, the membrane was incubated with secondary antibody diluted 1:7500 in blocking buffer (anti-rabbit-HRP, 1721019, and anti-mouse-HRP, 1706516, Bio-Rad). Signals were detected using Pierce ECL Western Blotting Substrate (Thermo Fisher Scientific, 32106). Densitometric quantitation of western blots was performed using Fiji software (National Institutes of Health) by an investigator masked to genotype. Signals from the protein of interest were normalized to actin levels.

### Statistical analysis

All experiments were performed at least three times (*n*≥3). Densitometric values were normalized to actin and log-transformed to stabilize the variance. To ensure that our data were suitable for Student’s *t*-test, we applied the Shapiro–Wilk and Kolmogrov–Smirnov tests for normality to our data. Both tests revealed that the majority of our data points (>90% in both tests) met the standard for normality, so we used unpaired two-tailed Student’s *t*-test to compare genotypes and establish the significance of our findings.

## Supplementary Material

10.1242/dmm.050858_sup1 Supplementary information
