# Supplementary material for: A Drosophila model for mechanistic investigation of tau protein spread
Source: Dis Model Mech. 2024 Oct 1;17(9):dmm050858. doi: 10.1242/dmm.050858 (PMC11463956; doi:10.1242/dmm.050858)
Supplement: Supplementary information [file dmm-17-050858-s1.pdf]

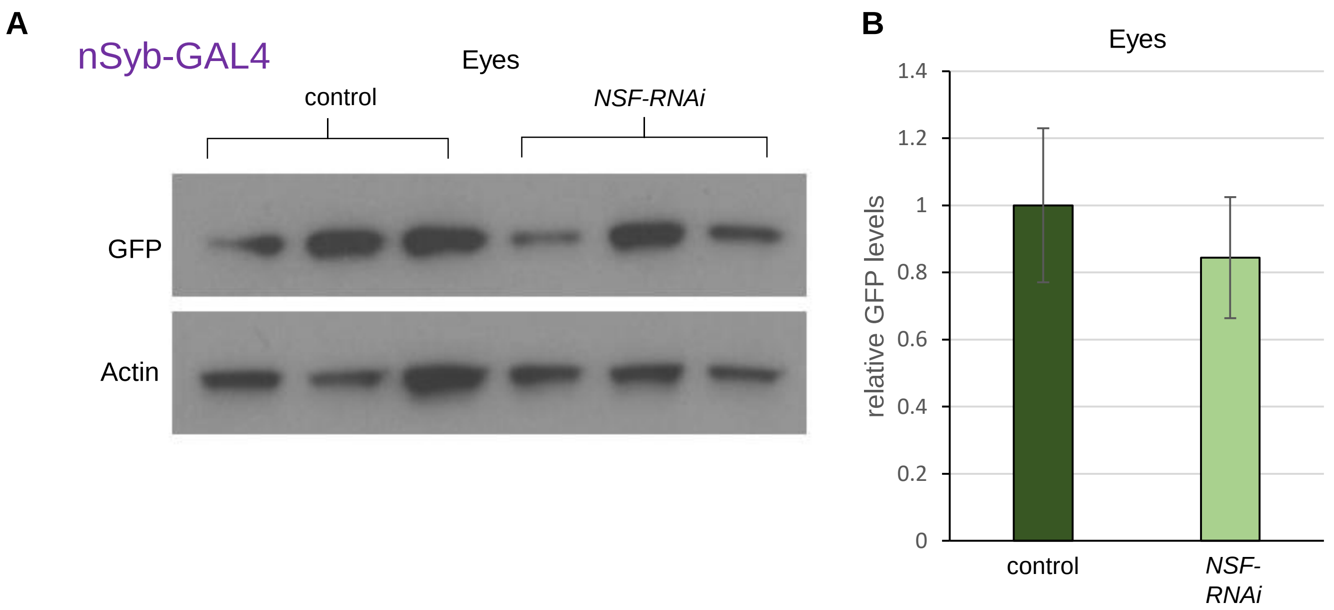

Fig. S1. GFP abundance is unchanged upon inactivation of NSF.(A) Western blot analysis of protein extracts from the eyes of 30-day-old flies expressing *tau-T2A-GFP* in the eye and an RNAi targeting *NSF* in neurons using anti-GFP. The experimental group bore *GMR-QF2w* driving *tau-T2A-GFP* and *nSyb-GAL4* driving a GAL4-responsive RNAi to *NSF*. Control flies lacked the RNAi transgene. Each condition was represented by three biological replicates. (B) Quantification of the data in panel A.

Disease Models & Mechanisms • Supplementary information
